# Host Cell–Virus Interaction 2.0: Viral Stratagems of Immune Evasion, Host Cellular Responses and Antiviral Counterattacks

**DOI:** 10.3390/v15081717

**Published:** 2023-08-10

**Authors:** Anupam Mukherjee, Parikshit Bagchi

**Affiliations:** 1 Division of Virology, ICMR-National AIDS Research Institute, Pune 411026, MH, India; 2 Department of Cell and Developmental Biology, University of Michigan, Ann Arbor, MI 48109, USA

As rightly stated by the author Mira Grant in her novel Countdown, “There is nothing so patient, in this world or any other, as a virus searching for a host”. Be it smallpox, influenza, or the coronaviruses, as history dictates, viruses have emerged and re-emerged to prove that something as small as being invisible to the naked eye is capable of causing human suffering. Through the years, viruses have evolved so as to see eye-to-eye with human evolution to exploit and survive within human host cells. It is simply commendable how deeply and clearly a virus understands its host cell, such that it interacts with it and convinces it to assist the virus in its pathogenesis. Therefore, an understanding of such host–virus interactions gives insight into the planning and development of antiviral strategies. 

With regard to the success of our previous issue on “Host Cell-Virus interaction”, we continued to receive some interesting articles from researchers all around the world, giving us an idea of the rigorous attempts being made globally to maneuver the interactions between the host and the virus, to ultimately help the body fight against an infection. Amongst the various articles received, 12 articles (5 research articles, 5 reviews, 1 comment, and 1 reply) outcompeted the others to make a place in this special issue of ours. As obligate intracellular parasites, the exploitation of the target cell for its own survival is of utmost necessity, and one of the ways in which it does so is by fighting against the host defense system. Our first article in the issue unveils a mechanism by which the Nipah virus disrupts the autocrine Interferon (IFN) signaling in the infected cells, emphasizing even more the role of inclusion bodies in Nipah infections, a study reported by Becker and Maisner [1]. 

Moving on to the findings reported by Kundharapu and Chowdary, which shed light on the structural discoveries pertaining to the non-structural proteins of the Dengue Virus (DENV), the advantages of bioinformatics have been adopted to uncover a plausible mechanism behind the enhancement of helicase activity of the DENV-NS3 protein [2].

The next article accepted for publication only points out the fact that, though HIV research has been holding its ground for a long time, it is far from reaching its near end. Here, Jin et al. emphasized the interaction of the human immunodeficiency virus (HIV)-1 Gag protein with the Multi-Aminoacyl-tRNA Synthetase Complex through the bi-functional human glutamyl-prolyl-tRNA synthetase (EPRS) subunit, discussing the effects of such an interaction on the virus and the host [3]. 

With Human Papilloma virus (HPV) research gaining ground, Rattay et al. proceed to state the results of their study on the HPV-8 proteins, E1 and E2, being responsible for the suppression of the innate receptor RIG-1-like MDA5, an event that might be crucial for HPV persistence in the host [4].

The next set of articles includes original research, a comment, and a reply pertaining to the research article, where Prezioso and their group account for the findings of their study on JC Poliomavirus (JCPyV) replication and microRNA expression after infection using archetypal and rearranged NCCR viral strains in COS-7 and SVGp12 cell lines. Their results highlighted the presence of viral miRNA in the exosomes of the infected supernatants, which could be carried on to uninfected cells, suggesting that the miRNAs may regulate the events of viral reactivation [5]. Following the publication of the research article, Henriksen and Rinaldo published a comment doubting the use of SVGp12 glial cells in the experiments as these cells are commonly infected with BK poliomavirus (BKPyV), providing evidence in support of their concern [6]. However, Prezioso et al. were kind enough to respond to these concerns, mentioning that all their assays were carefully screened via PCR amplification for the presence of the BKPyV genomes and confirmed that the SVGp12 cells used in their study were free from any BKPyV contamination, firmly stating that their results are precise and accurate [7]. In the next article, which is a review of the mechanisms of JCPyV entry into cells, Morris-Love and Atwood provide a descriptive account of the receptor-dependent and receptor-independent, extracellular vesicle-mediated entry of the virus into the target cells of the host in an attempt to explain virus tropism across the blood–brain barrier or cells that do not express the conventional receptors of JCPyV [8]. 

In another review, Dass et al. commendably describe the role of viral miRNAs and cellular miRNAs in human herpesvirus infections. With an elaborative take on the significance of the miRNAs in Herpes simplex viruses (HSVs), the authors continue to describe the regulatory effects of these miRNAs at different stages of HSV infection, such as replication and latency, while giving an account of the other 7 classes of herpesviruses. Also, the possibility of these miRNAs as biomarkers in herpesvirus infections has been discussed, along with their limitations [9].

With the advent of new technology such as CRISPR, genetic modifications that may be customized for genome-wide screens are being welcomed as a means to identify factors that participate in host–virus interactions. A review discussing the implementation of CRISPR for the study of genome-wide screens of Flaviviruses has been published by Kanojia et al., which marks the tenth article in this special issue [10].

In light of the fact that SARS-CoV-2 continues to wreck global health havoc, Sun et al. give a detailed account of the cellular sensors discovered and reported, along with the countermeasures undertaken by the virus to bypass or overpower these censors, in the fourth review of this special issue [11].

In the final article, which is also the last review article in the issue, Stephenson-Tsoris and Liang describe the proteins of both the host and the virus that contribute to the virus–host interaction events in the stages from entry until egress of the Hepatitis D Virus [12]. 

This special issue covers a diverse set of articles in terms of the techniques used, the approach to solving the question at hand, as well as the category of viruses. Even so, all the articles contribute to the understanding of virus–host interactions, which has been the prime objective of this second volume of the special issue. As Guest Editors of this Special Issue, we thank all the contributors to “Host Cell-Virus Interaction 2.0: Viral Stratagems of Immune Evasion, Host Cellular Responses and Antiviral Counterattacks”. We will be back soon with the third volume of this Special Issue with further interesting studies, cutting-edge research, and novel understandings on virus–host interactions and anti-viral drug discoveries.
